# Supplementary material for: The interactome of intact mitochondria by cross-linking mass spectrometry provides evidence for coexisting respiratory supercomplexes
Source: Mol Cell Proteomics. 2017 Dec 8;17(2):216–32. doi: 10.1074/mcp.RA117.000470 (PMC5795388; doi:10.1074/mcp.RA117.000470)
Supplement: Supplemental Data [file supp_RA117.000470_133922_0_supp_23978_fzffwf.zip › spectra_annotation/mito_spectra_annotation/303-1-10-1-21-1.pdf]

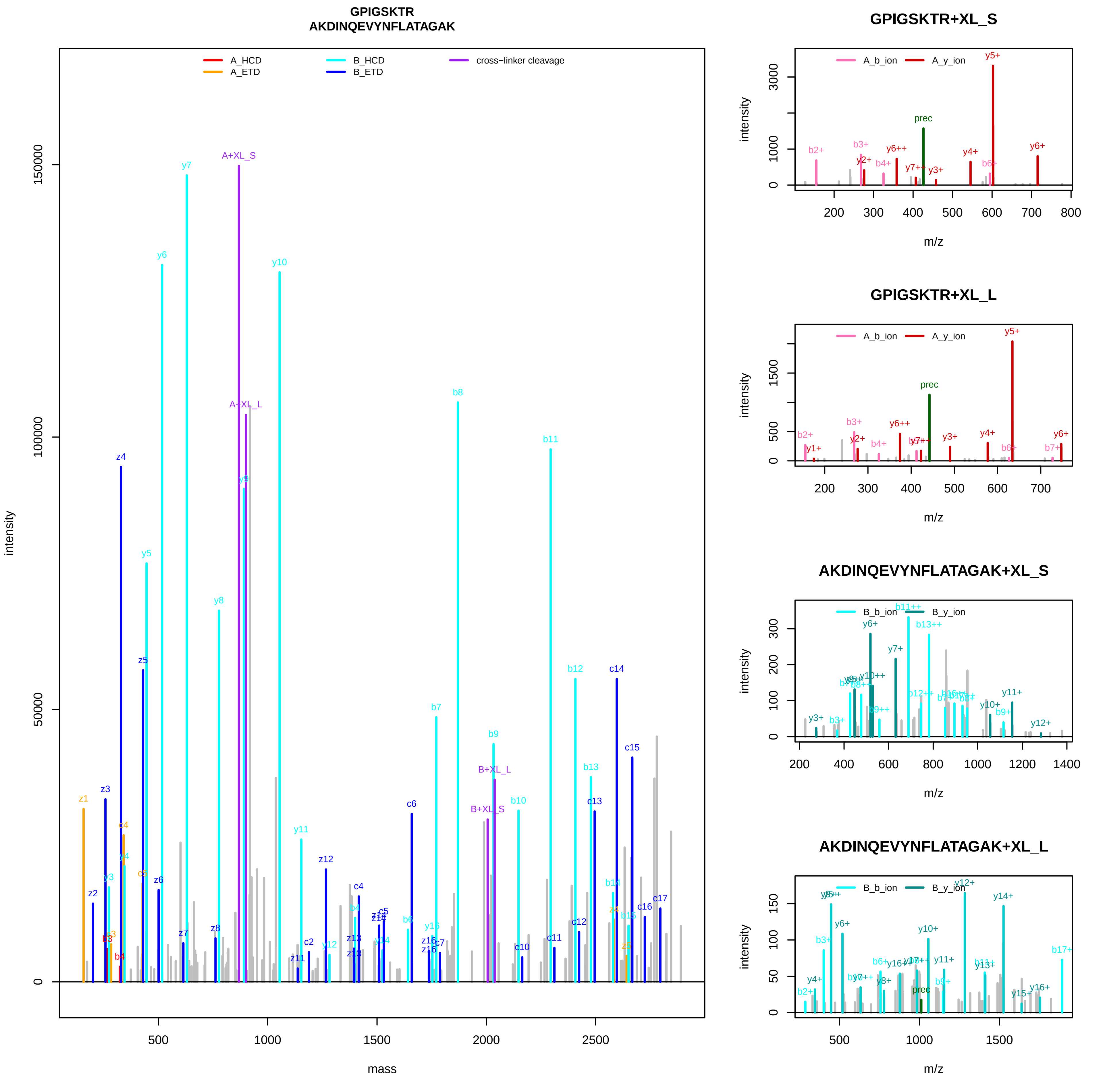

GPIGSKTR
AKDINQEVYNFLATAGAK
A_HCD
A_ETD
B_HCD
B_ETD
cross−linker cleavage
intensity
mass
0
50000
100000
150000
500
1000
1500
2000
2500
A+XL_S
A+XL_L
B+XL_L
B+XL_S
y7
y6
y10
b8
b11
y5
y8
y9
z4
z5
b7
b9
b12
c14
c15
b13
c13
b10
c6
z1
z3
z2
y3
c4
y4
c3
z6
y11
z12
c4
b4
z13
z14
c5
y14
b6
y15
z16
c7
c10
c11
c12
c16
c17
b14
b15
z4
z5
z7
z8
z11
c2
y12
b3
b4
y13
GPIGSKTR+XL_S
A_b_ion
A_y_ion
intensity
m/z
0
1000
3000
200
300
400
500
600
700
800
prec
y5+
b2+
b3+
y2+
b4+
y6++
y7++
y3+
y4+
b6+
y6+
GPIGSKTR+XL_L
A_b_ion
A_y_ion
intensity
m/z
0
500
1500
200
300
400
500
600
700
prec
y5+
b2+
y1+
b3+
y2+
b4+
y6++
y7++
y3+
y4+
b6+
b7+
y6+
AKDINQEVYNFLATAGAK+XL_S
B_b_ion
B_y_ion
intensity
m/z
0
100
200
300
200
400
600
800
1000
1200
1400
b11++
y6+
b13++
y7+
y10++
b12++
b9++
y3+
b3+
y10+
b9+
y11+
y12+
AKDINQEVYNFLATAGAK+XL_L
B_b_ion
B_y_ion
intensity
m/z
0
50
100
150
500
1000
1500
y12+
y14+
y6+
y10+
b3+
y4+
b2+
b6+
y16++
y17++
y11+
b9+
b11+
y13+
y8+
prec
y15+
y16+
b17+
